# 

**Published:** 1886-02

**Authors:** 


NOTICE TO OUR EXCHANGES. — Those of our Confreres who have not received the Transactions of the Texas State Medical Association for 1885, and desire a copy, can get it by dropping a postal card to us or to Dr. W. J. BURT, Sec. T. S. M. A.

EDITORIAL SUPPLEMENT

To DANIEL'S TEXAS MEDICAL JOURNAL, Feb. 1886, Vol. 1, No. 8.

---

HOMŒOPATHIC SURGERY (?) IN AUSTIN.

The following item is given us by Drs. Graves (City Physician) and Burt (Ex-City Physician). A negro man, Elgin, by name, was shot in the back, (pistol ball); paralysis of the lower limbs coming on, immediately, Homœopathic Surgeons being summoned, said that the ball had penetrated the abdominal cavity, and would be found lodged therein. In pursuance of this diagnosis, the homœopathic surgeons proceeded to *remove the twelfth rib* in search of it. After death, Drs. Graves, Burt, McLaughlin and Cummings found the ball lodged in the vertebral canal, severing the cord, verifying *their* ante-mortem diagnosis, as claimed.

---

We regret to learn of the death of Mrs. Paine, wife of our esteemed friend and correspondent, Dr. F. T. Paine, of Comanche, Texas, which occurred on the 7th, inst.

